# Assessing the functional impact of PfRh5 genetic diversity on ex vivo erythrocyte invasion inhibition

**DOI:** 10.1038/s41598-021-81711-9

**Published:** 2021-01-26

**Authors:** Adam J. Moore, Khadidiatou Mangou, Fatoumata Diallo, Seynabou D. Sene, Mariama N. Pouye, Bacary D. Sadio, Ousmane Faye, Alassane Mbengue, Amy K. Bei

**Affiliations:** 1 Department of Epidemiology of Microbial Diseases, Yale School of Public Health, New Haven, CT USA; 2 G4-Malaria Experimental Genetic Approaches & Vaccines, Pôle Immunophysiopathologie et Maladies Infectieuses, Institut Pasteur de Dakar, Dakar, Senegal; 3 Pôle Virologie, Institut Pasteur de Dakar, Dakar, Senegal; 4 Francis Crick African Network CAN Crick Fellow, London, UK

**Keywords:** Parasite biology, Parasite genetics, Parasite immune evasion

## Abstract

The PfRh5-Basigin ligand–receptor interaction is an essential step in the merozoite invasion process and represents an attractive vaccine target. To reveal genotype–phenotype associations between naturally occurring allelic variants of PfRh5 and invasion inhibition, we performed ex vivo invasion inhibition assays with monoclonal antibodies targeting basigin coupled with PfRh5 next-generation amplicon sequencing. We found dose-dependent inhibition of invasion across all isolates tested, and no statistically significant difference in invasion inhibition for any single nucleotide polymorphisms. This study demonstrates that PfRh5 remains highly conserved and functionally essential, even in a highly endemic setting, supporting continued development as a strain-transcendent malaria vaccine target.

## Introduction

Despite tremendous strides in malaria control, progress has plateaued and malaria is responsible for over 405,000 deaths and 228 million cases of disease globally each year, with the large majority of these cases and deaths being caused by the species *Plasmodium falciparum*^1^. The need for a highly effective second generation malaria vaccine that induces cross-strain protection remains as important as ever. One challenge in the creation of a highly effective vaccine is the extensive genetic diversity of *P. falciparum*. *P. falciparum* invasion of human erythrocytes is an essential stage of the life cycle and represents a stage amenable to targeting by both therapeutics and vaccines. Due to the essential nature of invasion for parasite survival, the merozoite form of *P. falciparum* uses an expanded repertoire of invasion ligands, which can be both variantly expressed and polymorphic, facilitating immune evasion and alternative receptor utilization^2^. While most of the invasion ligands involved in tight junction formation are dispensable and variant, *Plasmodium falciparum* reticulocyte binding protein homologous 5 (PfRh5) has been shown to be essential^3^. Antibodies targeting the PfRh5 receptor, basigin (BSG), are potently inhibitory across all laboratory adapted isolates from diverse geographic origins and a collection of short-term adapted *P. falciparum* field isolates^3^ and anti-PfRh5 antibodies have also been shown to be braodly neutralizing^4^. Vaccine-induced anti-PfRh5 antibodies from *Aotus* monkeys have been shown to be protective in a strain-transcendent manner in in vitro growth inhibition assays (GIA) and when naturally challenged with heterologous parasite genotypes^5,6^. Additionally, vaccine-induced antibodies in humans have been shown to sufficiently inhibit parasite growth across heterologous parasite genotypes in GIA assays^7,8^. A PfRh5 vaccine is currently in Phase I/IIa clinical trials.

While essential to erythrocyte invasion, PfRh5 is also highly conserved, with 16 nonsynonymous single nucleotide polymorphisms (SNPs) having been described in published data, and 35 in unpublished data from the Pf3k project (www.malariagen.net/pf3k)^9,10^. Of these, only five have been found at frequencies of 10% or more in any given population globally^11^. Antibodies raised against PfRh5 from the 3D7 reference strain have been shown to inhibit invasion across genetic variants in growth inhibition assays^4^. However, invasion assays with diverse field isolates are needed to discover novel SNPs and assess whether these genetic variations produce significant phenotypic impacts that significantly alter the invasion mechanism ex vivo. In the case of PfRh5, GIA assays have been shown to be a true mechanistic correlate of protection and so these assays, performed ex vivo with naturally occurring isolate populations, will be incredibly informative as to the degree of genotypic and phenotypic variation in the PfRh5-BSG pathway^12^. In this study, we investigate genotype–phenotype association studies ex vivo in a highly endemic region of Senegal to determine whether specific polymorphisms can influence the inhibition of monoclonal antibodies (mAbs) targeting the PfRh5-BSG invasion pathway.

## Results

**Figure 1:**
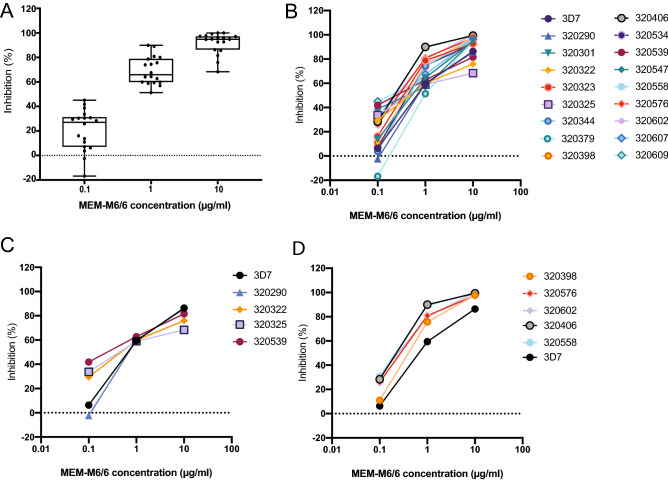
Monoclonal antibodies targeting BSG potently inhibit ex vivo *Plasmodium falciparum* isolates in a dose-dependent manner. The data from the 17 successful ex vivo invasion assays are shown here. PMR is calculated as: Final parasitemia (RPMI alone)/Initial parasitemia (RPMI alone). Invasion inhibition was calculated as 100- [(Average Percent invasion in anti-BSG MEM-M6/6 antibody wells)/(Average parasitemia in IgG1 isotype control wells) * 100]. Parasitemia was counted using a Miller reticle and 500 erythrocytes were counted for each assay. (**A**) Distribution of inhibition at each concentration: 0.1 $\mu$g/ml, 1 $\mu$g/ml, and 10 $\mu$g/ml, demonstrating overall conservation in inhibition at each concentration. Box and whisker plots show the median, interquartile range, and whiskers indicate the minimum and maximum. (**B**) Dose-response curves for inhibition with anti-BSG MEM-M6/6 antibody for each sample, including 3D7 for reference. (**C**) Dose-response curves for inhibition with anti-BSG MEM-M6/6 antibody for samples from the bottom 25% of inhibition (calculated from the 10 $\mu$g/mL concentration), including 3D7 for reference. (**D**) Dose-response curves for inhibition with anti-BSG MEM-M6/6 antibody for samples from the top 25% of inhibition (calculated from the 10 $\mu$g/mL concentration), including 3D7 for reference.

Thirty-one assays met the initial assay harvesting criteria of 95% rings (no more than 5% schizonts) within 96 h of starting the assay. Of these 31 harvested assays, 17 showed successful re-invasion with a PMR greater than or equal to 1. These 17 assays were analyzed and inhibition data is presented here (Fig. 1). We observed strong invasion inhibition across all 17 samples at 10 $\mu$g/ml of anti-BSG antibody. Antibody inhibition was dose-dependent (Fig. 1A), and was very similar to that of 3D7 (Fig. 1B). As antibody concentration decreased, there was more variability in the overall level of inhibition. This data implies that even in a highly endemic region of Senegal, ex vivo isolates remain highly sensitive to inhibition with monoclonal antibodies targeting the PfRh5-BSG pathway. We stratified samples into the lowest and highest quartiles according to inhibition at 10 $\mu$g/ml (Fig. 1C,D, Figure S1).

**Figure 2:**
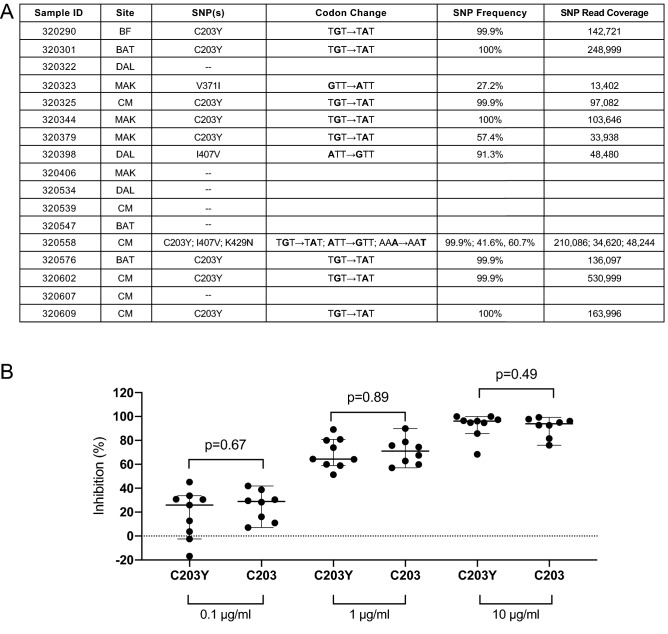
Genotype–phenotype associations identified that dominant SNPs in PfRh5 do not influence invasion inhibition. Next Generation Sequencing (NGS) analysis of samples from successful ex vivo invasion assays. (**A**) Description of the single nucleotide polymorphisms (SNPs) identified in patient samples from Kédougou. Specific site of collection is abbreviated as follows: Bandafassi (BF), Bantaco (BAT), Camp Militaire (CM), Dalaba (DAL), Mako (MAK). Both nucleotide and corresponding nonsynonymous amino acid changes are indicated. SNP frequency and read coverage at the SNP site (SNP Read Coverage) are also indicated. Sequences are accessible in Genbank (MW042085-MW042101). Of the 11 samples with SNPs at the dominant threshold (25%), 9 (52.9%) contained the C203Y SNP, 2 (11.8%) contained I407V, 1 (5.9%) contained K429N, 1 (5.9%) contained V371I. Sample 320558 is the only one to contain more than one SNP. Six samples contained no SNPs. These SNPs have been previously described in both published^9,11,13,19,20^ and unpublished data generated by the Pf3k project (www.malariagen.net/pf3k), representing 3,248 samples from 40 separate locations in 20 countries^9^. (**B**) Genotype–phenotype associations were performed for samples with the C203Y SNP and those without (C203Y vs C203 respectively), at each concentration of anti-BSG (MEM-M6/6) antibody. Medians and 95% confidence intervals are shown. No statistically significant difference between either genotype was observed at any of the antibody concentrations tested.

To determine whether specific SNPs may be associated with levels of inhibition, we identified dominant SNPs in these isolates through amplicon sequencing of PfRh5. Next Generation Sequencing (NGS) was performed on the 17 samples from the successful ex vivo assays. SNPs were called using a 25% “dominant” cut-off, reflecting a frequency likely to contribute to a potential measurable phenotypic difference. SNPs were found in 11/17 (64.7%) of the samples (Fig. 2A), relative to 3D7 reference. Ten samples contained a single SNP, while one sample (320558) had 3 SNPs. Of those with SNPs, 9 contained the C203Y SNP, 2 contained the I407V SNP, 1 contained the K429N SNP, and 1 contained the V371I SNP. Sample 320558 contained the SNPs C203Y, I407V, and K429N (Fig. 2A). Of note, while K429Q has been identified previously in West African populations (www.malariagen.net/pf3k)^9,10^, K429N has only been described once before, and also in Senegal^13^. A Mann-Whitney U Test found no statistically significant difference between the degree of invasion inhibition with anti-BSG mAb and the presence or absence of the C203Y SNP, for any concentration of antibody (Fig. 2B).

We established that this lack of a significant difference was not merely due to the inability of the sample size to detect a difference. Given the level of precision of the invasion inhibition assay, a meaningful difference in invasion inhibition (one for which we would be interested biologically) would be greater than 10%, and in practice, 20% or more. We have a sufficient sample size to detect a difference in inhibition of 20–23% with 80% power and an alpha level of 0.05. We also calculated Cohen’s d which is a measure of effect size, and our d values for 0.1 $\mu$g/ml, 1 $\mu$g/ml, and 10 $\mu$g/ml are 0.43, 0.13, and 0.13 respectively. These effect sizes would correspond to a small-medium difference in the means at 0.1 $\mu$g/ml, and a very small difference in means at 1 $\mu$g/ml and 10 $\mu$g/ml. If two groups’ means don’t differ by 0.2 standard deviations or more, the difference is considered trivial, even if significant. Taken together, the fact that we don’t detect differences in inhibition between the C203 and C203Y populations is due to the lack of a biologically relevant difference rather than a small sample size.

We next sought to determine whether some other measure of invasion efficiency or overall genetic diversity was associated with invasion inhibition. Using both linear regression and correlation coefficients on all data, as well as Mann-Whitney U Test to compare the extreme top and bottom quartiles of inhibition (Fig. 1C,D), we found that neither PMR, Parasitemia, nor number of SNPs was significantly associated with degree of inhibition (Figure S1B–D). Additionally, we observed that there was no significant difference in the overall number of SNPs or composition of SNPs in the 17 assays that met PMR quality criteria for inclusion compared to the 14 assays in which PMR was less than 1(Figure S2). Thus, while this population demonstrated a high prevalence of SNPs in PfRh5, there was no phenotypic association of genotype with invasion inhibition.

**Figure 3:**
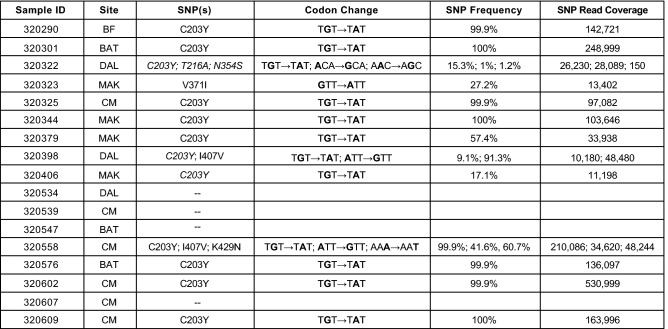
Novel SNPs in PfRh5 discovered through NGS at low frequencies. Next Generation Sequencing (NGS) analysis of samples from successful ex vivo invasion assays using a SNP discovery threshold of 1%. Description of the single nucleotide polymorphisms (SNPs) identified in patient samples from Kédougou. Specific site of collection is abbreviated as follows: Bandafassi (BF), Bantaco (BAT), Camp Militaire (CM), Dalaba (DAL), Mako (MAK). Both nucleotide and corresponding nonsynonymous amino acid changes are indicated. Italicized SNPs are those that were identified only with the discovery threshold (compare to Fig. 2). SNP frequency and read coverage at the SNP site (SNP Read Coverage) are also indicated. At this discovery threshold of 1% three samples (320322, 320379, and 320558) are shown to contain more than one SNP, with two of these samples (320322 and 320558) containing three SNPs. Most of these SNPs have been previously described in both published^9,11,13,19,20^ and unpublished data generated by the Pf3k project (www.malariagen.net/pf3k), representing 3248 samples from 40 separate locations in 20 countries^9^. Additionally, novel SNPs (T216A and N354S) were identified. These SNPs have not been previously reported in published or unpublished databases.

Further, by applying a more lenient “discovery” SNP calling threshold of 1% (variant coverage of at least 50 reads), we identified additional SNPs at low frequency in 2 additional samples (13/17 (76.5%), and the overall number of detectable SNPs per sample increased (Fig. 3). Some of these SNPs were novel SNPs (N75D, Q165R, T216A, K302R, F350L, N354S, R357G, and I420T), never yet described in other global populations (Fig. 3, Figure S3). This finding emphasizes the importance of conducting genomic diversity studies in highly endemic settings with complex samples, and also illustrates the power and utility of NGS at detecting minor alleles within the population.

## Discussion

This study shows that field isolates of *P. falciparum* exhibit similar levels of invasion inhibition as lab and culture-adapted isolates when presented with varying levels of anti-BSG monoclonal antibody, supporting the hypothesis that the PfRh5-BSG invasion pathway is essential in field parasite populations and that there is minimal phenotypic variation in utilization of this pathway. The inhibitory monoclonal antibody used in this study (MEM-M6/6) binds to the membrane proximal Ig domain D2 of basigin. We have shown the presence of the C203Y SNP, a SNP which is present at the PfRh5-BSG interface and is predicted to interact with the D2 of basigin^14^, does not have a significant impact on the parasites’ dependence on the PfRh5-BSG invasion pathway, as measured by inhibition with MEM-M6/6, in these ex vivo associative studies. While PfRh5 interacts with other parasite proteins as a part of a larger invasion complex, including PfRipr, PfCyRPA, and P113^15^, PfRh5 is the only member of this complex of proteins that binds to basigin directly^3,15^. However, recent studies have found that S197Y, which is also present at the PfRh5-BSG interface, contributes to immune evasion for a neutralizing monoclonal antibodies directed against PfRh5^7^. Our identification of the K429N SNP in Kédougou, previously described in Thiès, Senegal^13^, highlights potential region and even country-specific selective pressures that may arise in critical *Plasmodium* antigens and emphasizes the importance of conducting genotype–phenotype association studies in the field. The novel low frequency SNPs identified in this study will be of interest to pursue in terms of their function. Further studies combining genome editing to test the role of polymorphisms in an isogenic background would allow for precise investigation of whether genotypes observed in the field can alter inhibition with antibodies targeting the receptor–ligand interface. These results show PfRh5 phenotypic function and subsequent immune function are not impacted by diversity and the PfRh5-BSG invasion pathway remains essential in field samples. Previous work had shown similar results, but in lab strains and culture-adapted samples^3^. Here, we add the substantial evidence from ex vivo field samples from a highly endemic site, known to contain high levels of genetic diversity, exhibiting similar behavior. By demonstrating similar behaviors in field isolates that were observed in lab isolates, we add to the growing body of evidence that PfRh5 is an important, and functionally conserved target for a malaria vaccine.

This study has some limitations. First, the sample size of successful invasion assays is small (n = 17) for a genotype–phenotype association study. However, we have performed power calculations that indicate that we are adequately powered to detect a biologically relevant effect size, a difference in invasion inhibition of 20–23% or greater. An additional limitation of the study is that these ex vivo assays measure the phenotypic behavior of parasite populations within each patient (isolates), not individual parasite clones (strains). However, polyclonal infections are common in highly endemic regions, such as Kédougou and so this limitation may also be viewed as a strength in reflecting the reality of the field. Demonstrating that anti-BSG antibodies can significantly inhibit invasion across a genetically diverse parasite population provides a realistic idea of the potential success of a neutralizing antibodies from a PfRh5 vaccine in patients living in endemic regions.

An area for future directions is to understand at a deeper, more mechanistic level the contribution of specific SNPs to receptor binding and immune evasion, both the dominant SNPs as well as emerging novel SNPs found at low frequencies. In vitro protein–protein interaction assays have been previously performed for a subset of these dominant SNPs which are prevalent globally. Surface plasmon resonance (SPR) was performed with recombinant basigin as well as wild-type PfRh5 (from 3D7) and variants from 7G8 (C203Y) and GB4 (C203Y + I407V) to determine the binding kinetics and affinities^11^. Here, all variants had very similar biophysical binding parameters (Kd)^11^, indicating that these specific SNPs in PfRh5 do not show a dramatic difference in binding kinetics to basigin in vitro. These findings are in contrast to those evaluating the role of a rare basigin variant—E92K—the genetic determinant of the Ok$^{a-}$ blood group^3^. The basigin E92K variant had a two-fold lower affinity for PfRh5, due to slower association and a faster dissociation rate, and impacted *Plasmodium* invasion^3^. Our experimental future directions are to quantitatively test binding kinetics of each of the PfRh5 variants identified in this study. Our associative experiments in ex vivo isolates are important for identifying associations between invasion pathway and immune evasion; however, to conclusively show that any given SNP does not impact receptor binding or immune evasion, ideally each SNP would be introduced into an isogenic background using genome editing approaches (such as with CRISPR-Cas9), thereby specifically confirming the associations observed in the field. Taken together, our findings support the potential for strain-transcendent immunity generated in response to antibodies induced by the PfRh5 vaccine as thus far we have yet to identify any genotypes that escape potent neutralizing antibodies.

## Methods

### Study sites

This study was conducted with ethical approval from the National Ethics Committee of Senegal (CNERS) and the Institutional Review Board of the Yale School of Public Health. All research was performed in accordance with relevant guidelines and regulations, and informed consent was obtained from all participants and/or their legal guardians. Samples used in this study were collected as part of ongoing surveillance conducted by Institut Pasteur de Dakar investigating causes of febrile illness. Patients were recruited from five clinics in Kédougou, Senegal. Eligibility criteria for the main study was the presence of a fever (temperature greater than or equal to 38 degrees C) and/or a fever in the past 24 h. If a patient tested positive for *P. falciparum* on a *Pf*-specifc HRP2/3 rapid diagnostic test (RDT), a study clinical staffer assessed eligibility and obtained informed consent. A venous blood sample of 5ml in a EDTA vacutainer was obtained from consenting, enrolled patients and transported at room temperature from the clinic to the field lab for processing; no more than 6 h between draw and processing. Thin and thick blood smears were made for each sample to confirm mono-infection with *P. falciparum* by microscopy. Patient demographics and clinical parameters are described in Table S1.

### Ex vivo invasion assays

Initial parasitemia was calculated by thin smear microscopy for each sample. Samples were washed twice with unsupplemented RPMI media and resuspended in supplemented media. All cultures were plated with a starting parasitemia between 0.25%–1.0%, at 4% hematocrit with supplemented RPMI media. Samples with parasitemia greater than 1% were diluted to 1% parasitemia with uninfected O+ erythrocytes. Three concentrations of monoclonal anti-BSG MEM-M6/6 antibody (Abcam, ab119114) (10 $\mu$g/ml, 1 $\mu$g/ml, 0.1 $\mu$g/ml) were plated for each strain, alongside IgG1 isotype control (Abcam, ab81032), in duplicate, as previously described^3^. All antibodies used in the study were azide-free. The anti-BSG monolonal antibodies used in this study have been shown to specifically interrupt the interaction between PfRh5 and basigin in vitro by surface plasmon resonance (SPR)^16^. Additionally, wells were plated to allow for microscopic evaluation of assay progression and morphologic staging. Assay plates were cultured in a candle jar culture in a 37 degree C incubator. Assays were harvested when the smear-only control wells showed that at least 95% rings (no more than 5% schizonts remained) for 100 parasitized cells counted. If this criterion was not met, the culture was placed back into the incubator and smears were periodically made until it was ready to harvest. If a sample had not progressed past the schizont stage 96 h after starting the assay, it was discarded as it did not meet the criteria for a successful assay. Upon successful re-invasion, microscopy slides were made, stained with freshly diluted 10% Giemsa (Sigma-Aldrich) and counting was performed blinded. The parasitemia from the RPMI only wells were counted and averaged and these parasitemias were used to determine parasite multiplication rate (PMR). PMR is calculated as: Final parasitemia (RPMI alone)/Initial parasitemia (RPMI alone). Parasitemia from the duplicate test wells were averaged and invasion inhibition, relative to isotype controls, was calculated as follows: 100- [(Average Percent invasion in anti-BSG MEM-M6/6 antibody wells)/(Average parasitemia in IgG1 isotype control wells) * 100]. Of the n = 31 ex vivo invasion assays that had re-invaded and were harvested, the RPMI control wells were first counted by microscopy to determine if they reached a parasite multiplication rate (PMR) of at least 1; a quality control criteria for successful reinvasion. Seventeen of the 31 harvested assays showed a PMR of at least 1, and the inhibition data for these 17 assays are shown here.

### DNA extraction, amplification, and sequencing

DNA was extracted from dried blood spots (DBS) using QIAmp DNA Blood Mini Kit according to manufacturer’s instructions. PfRh5 exon 2 was PCR amplified using previously described primers^13^ and high fidelity polymerase. Size and purity of the PCR amplicon was confirmed on an agarose gel prior to Next Generation Sequencing (NGS). PCR amplicons for PfRh5 exon 2 were bead-purified (Omega), quantified by Qubit and Bioanalyzer, and normalized by KAPA qPCR and bioanalyzer. Library prep was performed using unique dual indexes (UDIs), samples were multiplexed, pooled, and sequenced with a NovaSeq with targeted coverage of 500,000 reads per sample at the Yale Center for Genome Analysis. Geneious Prime Software version 2020.0.5 (https://www.geneious.com) was used to analyze the sequencing data. Paired-end reads were trimmed with BBDuk plugin (https://www.geneious.com/plugins/bbduk; Adapter/Quality Trimming Version 38.37 by Brian Bushnell); trimmed sequences were mapped to the PfRh5 reference sequence from 3D7 (PF3D7 0424100). For dominant SNPs, we used a threshold of 25% of reads given minimum read coverage of 5000 reads. For SNP discovery, especially given a high complexity of infection among the samples, SNPs were called given a minimum frequency of 1% of all reads and coverage of minimally 5000 reads. Specific criteria for SNP calling were based on^17^ and were as follows: minimum quality score of Q35, found in both forward and reverse reads from each sequencing lane, minimum variant frequency of 25% (dominant) or 1% (discovery) and minimum coverage of 5000 reads, maximum variant P value of 0.0001, and minimum strand bias P value of 0.0005 when exceeding 65% strand bias. Final SNPs were independently validated by two individual researchers (A.J.M and A.K.B) and were further confirmed by visual inspection of individual mapped reads.

### Statistical analysis

GraphPad Prism 8.2.4 was used for all statistical analysis. We performed power calculations to determine whether the sample sizes in this study are enough to detect a biologically relevant effect. Based on previous precision estimates for GIA assays with multiple strains, an inhibition value within 10% would be within the limit of precision for biological replicate of a single genotype^18^. Specifically, the precision for replicates of 3D7 was calculated as 7.9% and precision for replicates of FVO is was 6.9%^18^. Given this, a meaningful difference in invasion inhibition (one for which we would be interested biologically) would be greater than 10%, and in practice, 20% or more. This study has 80% power to detect a difference in invasion inhibition of 20–23% or greater between two groups, with an alpha level of 0.05.

A Mann-Whitney U Test was performed to determine if mean inhibition was significantly different for isolates with mutation or without. Statistical associations between antibody-mediated invasion inhibition and PMR, parasitemia or number of SNPs were assessed using a linear regression model and Spearman nonparametric rank correlation coefficients (data not shown).

## Supplementary Information

Supplementary Information.
