# Supplementary material for: Assessing the functional impact of PfRh5 genetic diversity on ex vivo erythrocyte invasion inhibition
Source: Sci Rep. 2021 Jan 26;11:2225. doi: 10.1038/s41598-021-81711-9 (PMC7838290; doi:10.1038/s41598-021-81711-9)
Supplement: Supplementary file 1 — Supplementary Information. [file 41598_2021_81711_MOESM1_ESM.pdf]

Supplementary Information for

Assessing the Functional Impact of PfRh5 Genetic Diversity on Ex vivo Erythrocyte Invasion Inhibition

Adam J. Moore, Khadidiatou Mangou, Fatoumata Diallo, Seynabou D. Sene, Mariama N. Pouye, Bacary D. Sadio, Ousmane Faye, Alassane Mbengue, Amy K. Bei

Amy K. Bei.
E-mail: amy.bei@yale.edu

This PDF file includes:

Figs. S1 to S3
Table S1

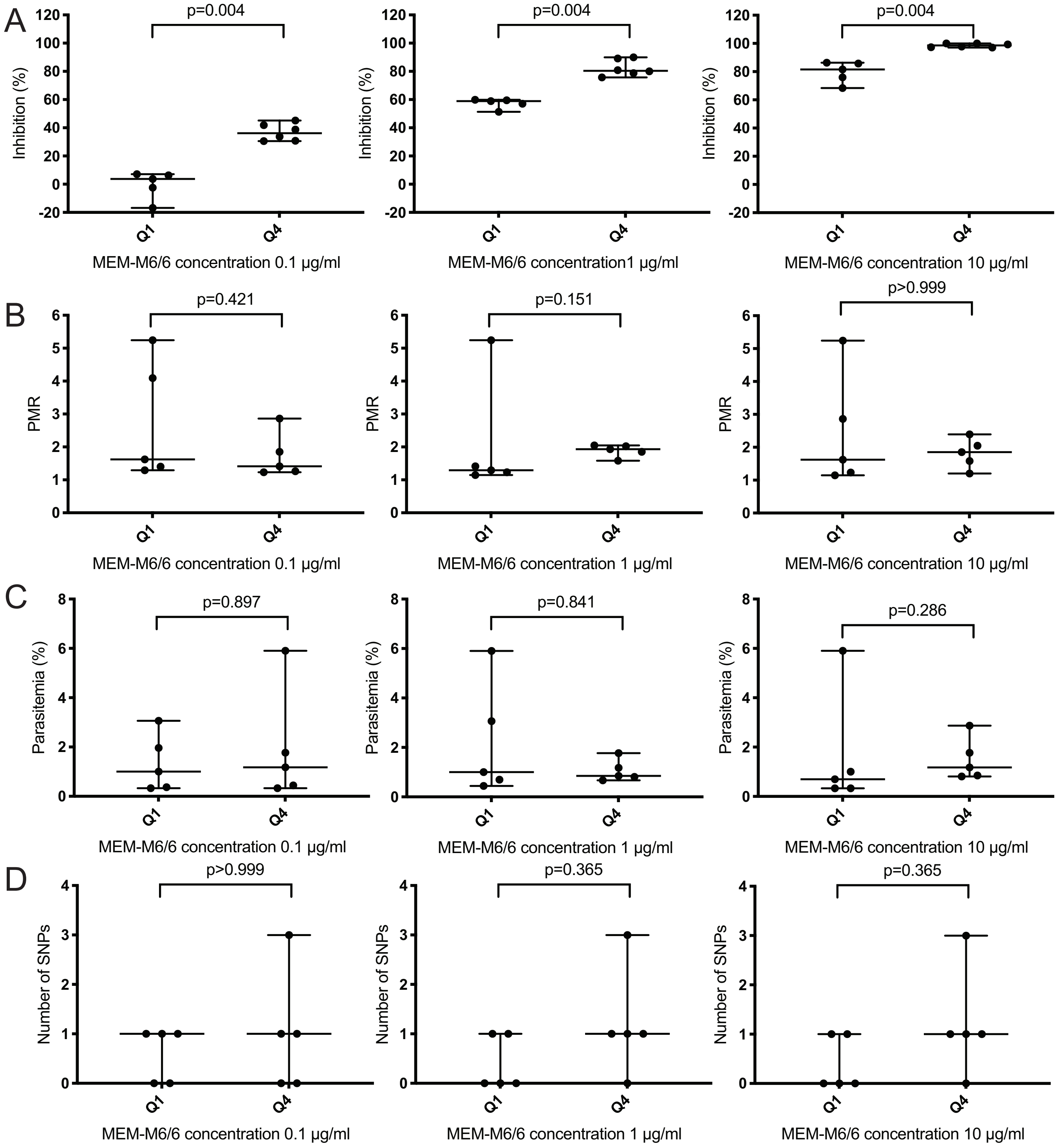

Fig. S1. PMR, Initial Parasitemia, and Number of SNPs do not influence inhibition with mAbs to BSG. Samples in Quartiles 1 (bottom 25%) and 4 (top 25%) of inhibition (A) for each antibody concentration level showed no statistically significant differences in Parasite Multiplication Rate (PMR) (B), Percent Parasitemia (C), or Number of Single Nucleotide Polymorphisms (D). Bars represent the medians with 95% confidence intervals.

A

Sample ID	Site	SNP(s)	Codon Change	SNP Frequency	SNP Read Coverage
320298*	BF	--			
320294	CM	C203Y	T**G**T→T**A**T	99%	105,016
320324	CM	C203Y	T**G**T→T**A**T	100%	91,360
320328	CM	C203Y; I407V	T**G**T→T**A**T; **A**TT→**G**TT	99.9%; 81.9%	96,744; 27,784
320336	MAK	H148D; C203Y; V371I	**C**AT→**G**AT; T**G**T→T**A**T; **G**TT→**A**TT	31%; 100%; 44.4%	5,983; 76,192; 13,734
320341	CM	C203Y	T**G**T→T**A**T	99.9%	93,913
320343	TOM	C203Y	T**G**T→T**A**T	99.9%	99,362
320345	MAK	--			
320346	MAK	I407V	**A**TT→**G**TT	74.9%	11,838
320364	TOM	C203Y	T**G**T→T**A**T	99.9%	174,264
320365	CM	--			
320393	BF	C203Y	T**G**T→T**A**T	99.9%	64,804
320564	CM	C203Y	T**G**T→T**A**T	99.9%	204,760
320603	CM	C203Y	T**G**T→T**A**T	99.9%	395,923

B

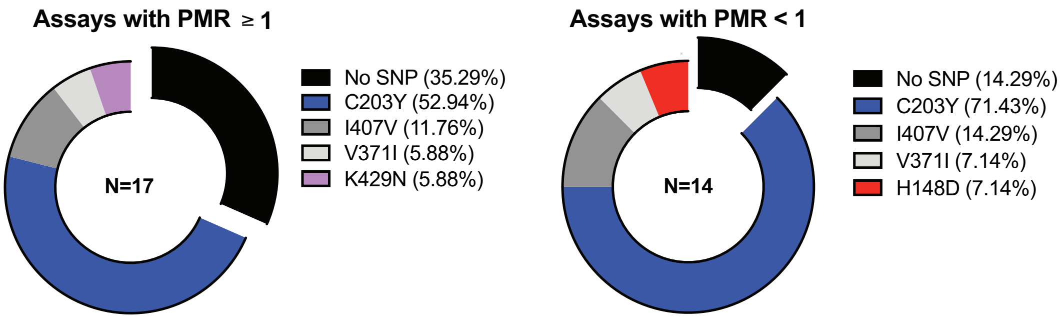

Fig. S2. ***Ex vivo*** **assays not meeting PMR threshold show similar distribution of SNPs at a dominant SNP threshold (25%).** Of the 31 invasion assays that were harvested, n=17 met the criteria for successful re-invasion of PMR greater than or equal to 1; whereas, n=14 *ex vivo* invasion assays that were harvested but for which PMR was less than 1. (A) Next Generation Sequencing (NGS) analysis was used to identify SNPs at the dominant threshold of 25% for which invasion assays were harvested but PMR<1. Specific site of collection is abbreviated as follows: Bandafassi (BF), Bantaco (BAT), Camp Militaire (CM), Dalaba (DAL), Mako (MAK). Both SNP and corresponding nonsynonymous amino acid changes are indicated. SNP frequency and read coverage at the SNP site (SNP Read Coverage) are also indicated. (B) Comparison between SNP frequencies in samples where PMR is > or = to 1 (n=17 Invasion inhibition Assays from Figure 1); left, and SNP frequencies in samples where PMR<1; right. Donut plots show the percentage of samples with no SNPs as well as with SNPs, the relative composition of each SNP among total SNPs, and the frequencies in parentheses show the frequency of any given SNP in the total number of samples.

A

Sample ID	Site	SNP(s)	Codon Change	SNP Frequency	SNP Read Coverage
320298*	BF	--			
320294	CM	C203Y	TGT→TAT	99%	105,016
320324	CM	C203Y; *K302R*	TGT→TAT; AAG→AGG	100%; 2.9%	91,360; 688
320328	CM	*N75D*; C203Y; *F350L*; I407V	AAT→GAT; TGT→TAT; TTC→CTC; ATT→GTT	1.7%; 99.9%; 2.2%; 81.9%	141; 96,744; 540; 27,784
320336	MAK	H148D; C203Y; V371I	CAT→GAT; TGT→TAT; GTT→ATT	31%; 100%; 44.4%	5,983; 76,192; 13,734
320341	CM	C203Y	TGT→TAT	99.9%	93,913
320343	TOM	C203Y	TGT→TAT	99.9%	99,362
320345	MAK	*C203Y*	TGT→TAT	23.7%	5,854
320346	MAK	*Q165R; C203Y; T216A*; I407V	CAA→CGA; TGT→TAT; ACA→GAC; ATT→GTT	1.0%; 10.5%; 1%; 74.9%	92; 2,559; 241; 11,838
320364	TOM	C203Y	TGT→TAT	99.9%	174,264
320365	CM	--			
320393	BF	C203Y; *R357G*	TGT→TAT; AGA→GGA	99.9%; 1.6%	64,804; 187
320564	CM	C203Y	TGT→TAT	99.9%	204,760
320603	CM	C203Y	TGT→TAT	99.9%	395,923

B

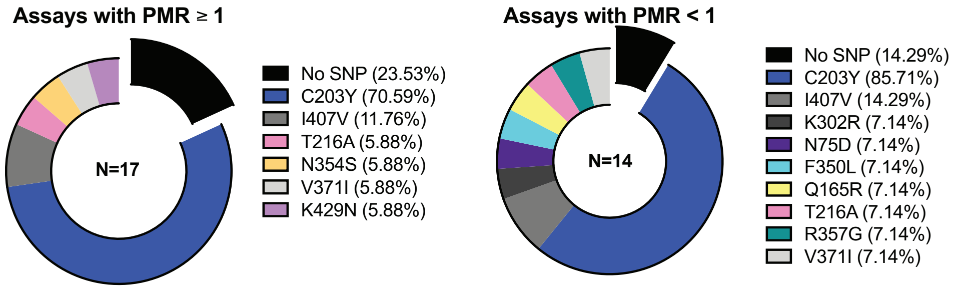

Fig. S3. *Ex vivo* assays not meeting PMR threshold show similar distribution of SNPs at a discovery SNP threshold (1%). Of the 31 invasion assays that were harvested, n=17 met the criteria for successful re-invasion of PMR greater than or equal to 1; whereas, n=14 *ex vivo* invasion assays that were harvested but for which PMR was less than 1. (A) Next Generation Sequencing (NGS) analysis was used to identify SNPs at a low frequency, namey the discovery threshold of 1% for which invasion assays were harvested but PMR<1. Specific site of collection is abbreviated as follows: Bandafassi (BF), Bantaco (BAT), Camp Militaire (CM), Dalaba (DAL), Mako (MAK). Both SNP and corresponding nonsynonymous amino acid changes are indicated. SNP frequency and read coverage at the SNP site (SNP Read Coverage) are also indicated. (B) Comparison between SNP frequencies in samples where PMR is > or = to 1 (n=17 Invasion inhibition Assays from Figure 1); left, and SNP frequencies in samples where PMR<1; right. Donut plots show the percentage of samples with no SNPs as well as with SNPs, the relative composition of each SNP among total SNPs, and the frequencies in parentheses show the frequency of any given SNP in the total number of samples. With this discovery threshold, we identified novel SNPs not previously described, namely: N75D, Q165R, T216A, K302R, F350L, N354S, R357G, and I420T.

Sample	Site	Age, years	Sex	Temperature,ºC	Initial Parasitemia [a] (%)	Adjusted Parasitemia[b] (%)	Re-Invasion Parasitemia [c] (%)
320290	BF	36	F	38	0.33	0.33	0.53
320301	BAT	12	M	36	3.02	1.00	1.94
320322	DAL		F	38.5	0.70	0.70	1.15
320323	MAK	10	F	39.8	0.67	0.67	1.35
320325	CM	12	M	38.4	5.90	1.00	1.23
320344	MAK	5	F	39.3	1.96	1.00	4.09
320379	MAK	17	M	38.8	3.06	1.00	1.29
320398	DAL	10	M	35.7	2.87	1.00	1.20
320406	MAK	18	M	38.6	0.85	0.85	1.75
320534	DAL	35	F	39.6	0.37	0.37	0.52
320539	CM	18	F	36.2	0.33	0.33	0.95
320547	BAT	30	M	37.8	0.44	0.44	0.62
320558	CM	12	F	39.5	1.18	1.00	1.85
320576	BAT	2	F	37	0.81	0.81	1.93
320602	CM	12	F	39.9	1.77	1.00	1.58
320607	CM	18	F	38.7	0.54	0.54	1.75
320609	CM	23	M	38	1.77	1.00	1.26

Table S1. Patient Demographics. Demographic data from patients enrolled in this study for which genotype-phenotype associations were performed. De-identified sample IDs (Sample), collection site (Site), Age (in years), Sex, Temperature, Initial parasitemia, Adjusted parasitemia, and Final parasitemia. Sample distribution by site was as follows: Bandafassi (BF) 1(5.9%), Bantaco (BAT) 3(17.6%), Dalaba (DAL) 3(17.6%), Mako (MAK) 4(23.5%), Camp Militaire (CM) 6 (35.3%). All patients were *P. falciparum* positive by a *Pf*-specifc HRP2/3 rapid diagnostic test (RDT) and confirmed to be infected with *P. falciparum* only, harboring no mixed-species infections by microscopy.

a - Initial parasitemia is the percentage of infected cells at enrollment, when counting 4500 total erythrocytes by Miller reticle

b - Adjusted parasitemia is the parasitemia adjusted prior to invasion assay. Samples with initial parasitemia greater than 1% were diluted to 1% with uninfected O+ erythrocytes

c - Re-invasion parasitemia is the percentage of infected cells after assay harvest in the RPMI only (no antibody) wells, when counting 4500 total erythrocytes by Miller reticle. Sextuplicates were averaged.
